# Effects of motor imagery training of Parkinson's disease: a protocol for a randomized clinical trial

**DOI:** 10.1186/s13063-019-3694-8

**Published:** 2019-11-09

**Authors:** Isaíra Almeida Pereira da Silva Nascimento, Lorenna Marques de Melo Santiago, Aline Alves de Souza, Camila de Lima Pegado, Tatiana Souza Ribeiro, Ana Raquel Rodrigues Lindquist

**Affiliations:** 1 Department of Physical Therapy, Federal University of Rio Grande do Norte, Campus Universitário Lagoa Nova, Senador Salgado Filho Avenue, 3000, PO Box 1524, Natal, Rio Grande do Norte 59078-970 Brazil; 2 Anita Garibaldi Center for Education and Research in Health, Santos Dumont Institute, RN 160 Highway, Km 1,5, N° 2010, Jundiaí District, Macaíba, Rio Grande do Norte 59280-970 Brazil

**Keywords:** Parkinson’s disease, Rehabilitation, Cerebral activation, Neurological gait disorders

## Abstract

**Background:**

Gait disorders in individuals with Parkinson’s disease (PD) may be associated with alterations in the motor control system and aggravated by psychoemotional and cognitive issues. Therapeutic strategies aimed at self-perception and motor regulation seem to be promising. Motor imagery (MI) has been shown to be one of these strategies, but there is still no clear evidence of its applicability in this population. The aim of this trial is to determine the effects of motor-imagery training on the gait and electroencephalographic activity of individuals with PD.

**Methods/design:**

The sample will consist of 40 individuals, aged between 45 and 75 years, in the mild and moderate phase of the disease, with the ability to generate voluntary mental images. They will be assessed for cognitive level, degree of physical disability, mental-image clarity, kinematic gait variables, electroencephalographic activity and mobility. Next, subjects will be randomly assigned to an experimental group (EG) and a control group (CG). The EG will perform motor imagery and gait, while the CG will only engage in gait exercises. Twelve training sessions will be conducted lasting up to 90 min each, three times a week, for 4 weeks. The subjects will be reassessed on the kinematic variables of gait, electroencephalographic activity and mobility at 1, 7 and 30 days after the final training session.

**Discussion:**

The results may provide an important advance in neurological rehabilitation where an easy-access and low-cost intervention may help to improve gait, electroencephalographic activity and mobility in individuals with PD.

**Trial registration:**

Clinicaltrials.gov, ID: NCT03439800. Registered on 15 November 2017.

**Electronic supplementary material:**

The online version of this article (10.1186/s13063-019-3694-8) contains supplementary material, which is available to authorized users.

## Background

Motor alterations caused by Parkinson’s disease (PD) significantly affect gait, creating cognitive dependence in improving movement patterns; that is, gait becomes more vulnerable to external influences [1]. External clues provide information and require the individual’s attention to movement, activating frontal cortex regions to control its execution [2–4]. Studies suggest that these clues may help improve gait pattern and electroencephalographic (EEG) activity in patients with PD [5, 6]. A recent review study conducted by Bočková and Rektor (2019) [7] indicates that patients with PD face a general slowdown of background activity, excessive synchronization of beta-rhythm activity and disturbed movement-related gamma-rhythm oscillations in the basal ganglia and in the cortico-subcortical and cortico-cortical motor loops, suppressible by dopaminergic medication, as well as by high-frequency deep-brain stimulation. Although dopaminergic therapy is currently the best treatment for PD, gait dysfunctions are commonly resistant to drug therapy, especially in the advanced stages of the disease.

Motor imagery (MI) [8], defined as imagining a motor action without physically executing it [9], is a cognitive strategy that, along with other types of external clues, demands attention to the sequence of a trained movement, which can be performed visually or kinesthetically. It has been shown that MI can produce replicable EEG patterns over primary sensory and motor areas [10, 11], and despite deficits in the supplementary motor area from the indirect effect of the basal ganglia, patients with PD have preserved locomotor imagery observed during the on-medication state [12]. The mesencephalic locomotor region (MLR), located in the brainstem region and consisting of the pedunculopontine and cuneiform nuclei, is modulated by changes in imagined locomotion in healthy humans [13]. MLR also modulates cortical networks similar to those involved during real gait [14].

In neurological rehabilitation, the use of MI has generally been associated with physical practice (PP). Motor imagery has several advantages, such as the opportunity to increase the number of repetitions safely and autonomously, without excessive fatigue, in addition to allowing the mental training of motor tasks, when and where the patient wants or is able to perform them. Furthermore, MI enables more demanding or complex motor tasks, such as gait, or when PP is impossible or very difficult. Despite these advantages, MI is a complex process that is not easy to integrate into clinical practice [15]. Its association with PP, however, seems to be more effective than PP alone in enhancing motor function [16].

Two studies that associated MI with PP, in order to determine the motor effects on individuals with PD, showed a significant decline in bradykinesis [17] and an improvement in the kinematic aspects of gait [18]. Another study that correlated MI with PP showed no gait improvement in individuals with PD [19]. A fourth study that compared MI with relaxation sessions, both associated with PP, found no improvement in the mobility of these patients [20]. The protocols used, different training times between studies and the lack of follow-up precluded confirming the duration of the effects observed.

In light of the gaps in the methodologies presented, a therapeutic plan can be devised involving MI, which has been shown to be promising, albeit still inconclusive. As such, there is a need to develop more effective training protocols in order to optimize the rehabilitation process of individuals with PD. To that end, a new MI protocol associated with gait was created for patients with PD, based on the protocols of El-Whishy [18] and Santiago [19], but differing in terms of training time, observation method and follow-up.

Few PD studies have investigated EEG functions during gait activity. Therefore, this study aims to contribute to the literature on the pattern of brain activation (coherence intra- and inter-hemispheres and the spectral power of alpha and beta rhythms) of individuals with PD during gait activity, before and after the MI intervention. Clinical quantitative measures of gait and mobility, most commonly investigated by other studies, will also be analyzed and correlated with EEG data.

Thus, this randomized clinical trial protocol should determine the effects of MI training on the gait and EEG activity of individuals with PD.

## Methods/design

### Design

**Fig. 1:**
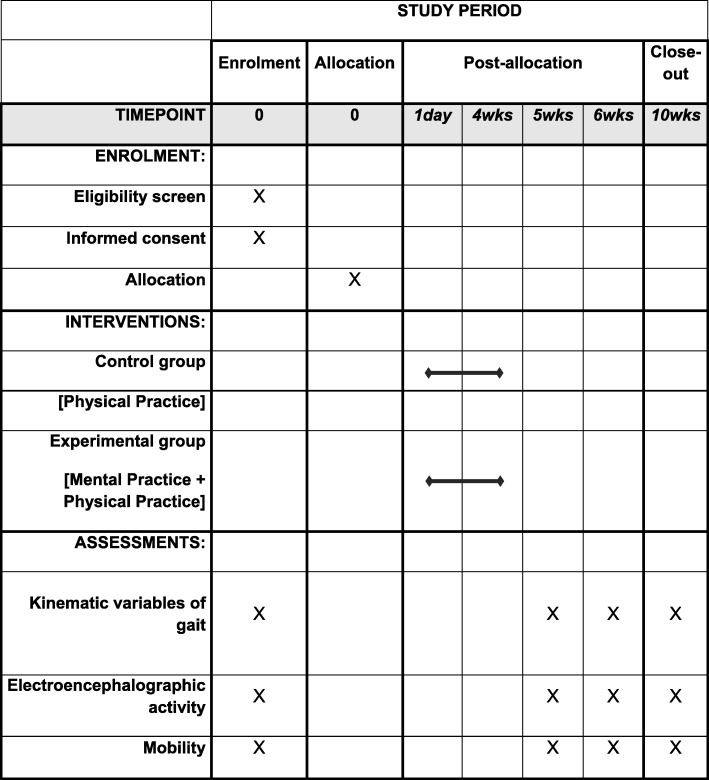
The schedule of enrollment, interventions and assessments demonstrated in the Standard Protocol Items: Recommendations for Interventional Trials (SPIRIT) Figure

This is a single-blind, randomized controlled clinical trial, in line with the Standard Protocol Items: Recommendations for Interventional Trials (SPIRIT) guidelines (Fig. 1). It will be conducted at the Laboratory of Intervention and Analysis of Movement (LIAM), in the Physical Therapy Department of the Federal University of Rio Grande do Norte (UFRN). Participants will be recruited from a population of patients with PD treated in public and private hospitals and reference centers in the city of Natal, Rio Grande do Norte state, Brazil. They will be randomly allocated to an experimental group (EG; MI associated with PP) or a control group (CG) (only PP). Outcome measures will be collected by trained researchers at the start (week 0) and at the end of the intervention (weeks 5 and 6), and 1 month after the intervention (week 10). Analyses of inclusion criteria, obtaining informed consent, data collection and statistical analyses will be carried out by the researchers, who will be blind to group allocation. Participants will be evaluated and advised of the study procedures, in addition to giving their informed consent. This project was approved by the Research Ethics Committee of the Federal University of Rio Grande do Norte, under protocol number 2.057.658 and registered as a clinical trial at www.ClinicalTrials.gov (ID: NCT03439800).

### Sample size estimates

The sample size was calculated using the OpenEpi public domain program, version 3.01 [21]. The calculation, based on the outcome variable “hip range of motion” of individuals with PD submitted to MI associated with the PP of gait, obtained by El-Whishy et al. [18] A power of 80% was considered as well as a 95% confidence interval. The mean and standard deviations were 54.7° and 7.2°, respectively, from which the sample size of 34 individuals (17 in each group) was obtained. Added to this value was 10% for possible sample losses, resulting in a final sample of 40 individuals, 20 each in the two groups under study.

### Participants

#### Inclusion and exclusion criteria

Patients will be included according to the following criteria: (1) Diagnosis of PD by a neurologist; (2) Age between 45 and 75 years; (3) In the mild to moderate phase of the disease (including patients between stages 1.5 and 3 on the Hoehn and Yahr scale) [22]; (4) Using antiparkinson medication, without adaptation during study participation; (5) Exhibiting no cognitive impairment, according to the Mini-Mental State Examination (MMSE). The cutoff point was established considering the individuals’ schooling (illiterate: 18; some schooling: 24) [23]; (6) Be able to imagine motor activities in the kinesthetic modality (according to the Revised Movement Imagery Questionnaire – MIQ-R). The cutoff point was 20 for the kinesthetic modality, indicating at least “somewhat easy to feel” the kinesthetically imagined movement [24]; (7) Not having undergone stereotaxic surgery; and (8) No other associated neurological diseases; no musculoskeletal alterations that hinder gait; corrected vision or hearing. The following patients will be excluded: (1) Those exhibiting hemodynamic instability before or during training (systolic and diastolic blood pressure above 180 mmHg and 110 mmHg, respectively) [25]; (2) Not understanding any stage of the training protocol; and (3) Experiencing acute pain and/or discomfort that would hinder the proposed activities.

### Outcomes

#### Primary outcome

The hip range of motion, a kinematic variable of gait, will be obtained by the Qualisys Motion Capture System (Qualisys Medical AB, 411 13, Gothenburg, Sweden). This system records the spatio-temporal variables of gait, as well as the angular variations of the hip, knee and ankle joints. It is a photographic system based on a video that reconstructs movement in three dimensions (3D), composed of six Qualisys Oqus 300 cameras, connected in series and that emit and capture infrared light, which is reflected by spherical markers placed on specific anatomical structures.

#### Secondary outcomes

The secondary outcomes will be the spectral power of the alpha and beta rhythms, intra- and inter-hemisphere coherence between frontal and fronto-central channels and mobility. Electroencephalographic activity will be measured by Emotiv EPOC+, which provides EEG data. The device uses contact sensors attached to the flexible plastic arms of a wireless headset. The headset has 14 sensors that are able to detect facial expressions, head rotation, emotions and conscious mental commands. For the present study, the changes in EEG activity captured on the surface of the patient’s head will be monitored before and after 12 training sessions (including follow-ups). MATLAB software will be used to process the data [26, 27]. Mobility will be assessed by the Timed Up and Go Test (TUG). This consists of rising from an ordinary chair (with no arms), walking 3 m, turning through 180°, and sitting down again as fast as possible wearing typical walking shoes. The shorter the time to perform the activity, the better the mobility [28]. The time spent on the two attempts will be recorded using a digital stopwatch.

### Randomization

Randomization will be computer-generated in randomized blocks using the randomization.com system. The process will be carried out by a volunteer not affiliated with the research, who will preserve allocation anonymity, randomly separating the individuals into a control group (CG) and experimental group (EG). The volunteer will also prepare sealed envelopes, using codes to represent the groups.

Only the researchers in charge of conducting the training sessions will be aware of meaning of each code and participant allocation. They will open the envelope corresponding to the patient number before patients start the training.

The researchers responsible for the initial assessment and reassessment will not be informed of allocation during data collection and statistical analysis. Data analysis will also be performed by one of the evaluators, who will only have access to the codification and will not be informed about which group each code corresponds to.

The present protocol has been prepared in accordance with relevant items from the SPIRIT Checklist (see Additional file 1) and the SPIRIT Figure (Fig. 1).

### Intervention

This protocol will be based on those of El-Whishy [18] and Santiago [19]. Training will start on the day following the initial assessment, which will be divided into 2 days.

The subjects who are randomly allocated to the EG will be submitted to a protocol consisting of five training stages, as follows: awareness, problem identification, progressive relaxation, MI of the task and PP of the task. The CG will undergo the following stages: awareness, problem explanation, progressive relaxation and PP of the task; that is, stages 1, 2, 3 and 5.

After opening the identification envelope, the researcher in charge of training will describe the stages that the participants will be submitted to. Before this, blood pressure and heart rate will be measured and monitored by a digital sphygmomanometer (Visomat Comfort III, Incoterm*®,* São Paulo, Brazil).

In the event that patients exhibit altered vital signs, they will be asked to remain seated and try to relax. If the situation persists, the patient will be instructed to visit their physician.

Before training sessions begin, the researcher in charge will ask the patient to walk around the course to be used that day if it is the first, fifth or ninth session, so that the execution time can be recorded and used during the fourth stage. A detailed description of the training protocol is shown below.

### EG and CG protocol

#### Awareness

In the EG, the subjects will analyze the sequence of the gait cycle, in order to understand the gait phases, which should facilitate motor planning and problem identification. The therapist will show a video of a typical normal gait for an adult man or woman with no pathologies and will compare it to the video of the patient’s own gait. The patient will watch the videos in two planes: the coronal and sagittal, which will be shown more than once. After viewing both videos, the patient will be encouraged to analyze and score their own gait characteristics, which differ from those of the individuals with no pathology. If the subject cannot adequately verbalize, the therapist can give hints on which important aspects should be observed. The CG subjects will watch a video on PD that does not mention physical therapy treatments for gait. The time allotted to watch the video will be the same as for the EG.

#### Problem identification/explanation

In the EG, the subjects will identify gait problems and compare their walking and typical gait. Next, they will use comparative information for feedback. It may be necessary to encourage the patient in terms of the characteristics that should be compared, such as arm balance, stride length and gait speed. In the CG, the patients will explain their understanding of the video that they watched in the previous stage.

#### Progressive relaxation

At the start, both groups’ participants will be instructed to sit comfortably in a chair with their backs supported, hands in their lap, close their eyes while breathing slowly and deeply through the nose and say the word “hoo” silently to themselves while exhaling. They will be instructed to concentrate and be aware of their breathing, while repeating the respiratory pattern 10 times.

#### MI of gait

In the first part, the EG subjects will be asked to mentally repeat from stage 1 to the problem-identification stage, with their eyes closed. After the mental-imagery phase, subjects will be asked to verbalize a difference between normal gait sequences and their own gait. In the second part, they will practice MI of the sequence of movements of the gait pattern in adults with no pathologies (considered the typical pattern), imagining themselves walking along a straight course and feeling all the components of gait (for example, movement, muscle action, arm balance and weight bearing), while correcting the problems found in the video. Seated and with their eyes closed, they will imagine each movement stage in the kinesthetic modality, where “feeling” must be emphasized: the movement, muscle action, arm balance, weight-bearing, etc.

During the EG stage, the setting should be as quiet as possible and the patient will be asked to wear noise-canceling earphones. There will be three sessions of 10 repetitions. Each repetition will be controlled and last the same amount of time as that measured during the patient’s lap around the course, with 20 s rest between each repetition. At each repetition, patients will be instructed to close their eyes and imagine the sensation present while rising from a chair, adopting their posture and when walking around the course. They must remember to start with the least affected limb in order to perform the gait pattern, making the expected corrections. At the end of each repetition, the patient will receive a sign from the therapist, who will provide instructions and time the duration of the next repetition.

Each MI session will be held alternately with the PP sessions (described in the next topic: “stage 5 PP of gait”). MI will always be prior to the PP of gait.

#### PP of gait

Both groups will engage in the PP of gait, paying close attention to the sequence. This will be performed on a flat, firm, 6-m-long surface, following the same number of series and repetitions as in the second part of MI. In the EG, the second part of MI and PP will be repeated alternately. In the CG, execution will occur normally, without alternate repetitions of stages 4 and 5, but there will also be guidelines on the PP stages to be performed.

In the first of 12 sessions, during stage 5, the patient will be asked to walk around the course as best they can. During the repetitions, the researcher can verbalize a number of feedback sentences, in relation to the aspects of gait that should receive attention and be corrected. This will be allowed until the second session. From the third session onwards, at the onset of PP, patients will be reminded about the corrections previously highlighted.

From the fifth session onwards, EG subjects will undergo the previously described protocol; however, in stage 4, the imagined gait will be in a setting with obstacles, represented by a busy street. The patient will be instructed to imagine walking along the street, dodging people, avoiding holes, walking down sidewalks and entering stores with narrow doorways. In stage 5, they will perform gait with obstacles, as follows: zigzagging around two cones, walking through a narrow doorway, climbing up and down one step, climbing up and down one ramp, climbing up and down one step, stepping over three mini barriers and one small box, climbing up and down one small ladder, walking over foam and then returning over the entire course.

In the first repetition of the fifth session in the PP portion, the obstacles will be arranged in the same way as shown in the videos, and the patient will start at the beginning of the circuit. At each series conducted, the point where the patient starts the trajectory will be changed and this pattern will be used for the others. In the subsequent sessions, the course will be slightly changed and based on photographs and the same change will be repeated with all the patients. The CG will also perform the aforementioned protocol, but in stage 5, gait will be executed in an environment with obstacles (the same as the EG).

From the ninth training session onwards, EG subjects will carry out both MI and PP of gait with two tasks. Mental imagery will involve a supermarket, where the patient will be instructed to imagine walking around the environment, shopping for products beginning with a letter that will be drawn at each series.

The dual task performed in stage 5 will be different at each session, progressing in difficulty. In the ninth session, the patient must say words starting with the letters drawn in each series; in the 10th session, they will be asked the names of fruits according to the letters selected; in the 11th session, it will be the names of animals and, finally, in the 12th session they will perform subtractions, starting with a decreasing sequence of 90 minus 3. Furthermore, another motor activity will be associated with gait such as picking up an object along the way and bringing it back to the start of the course. In the CG, subjects will only engage in dual-task gait, following the same protocol of activities established for the EG.

**Fig. 2:**
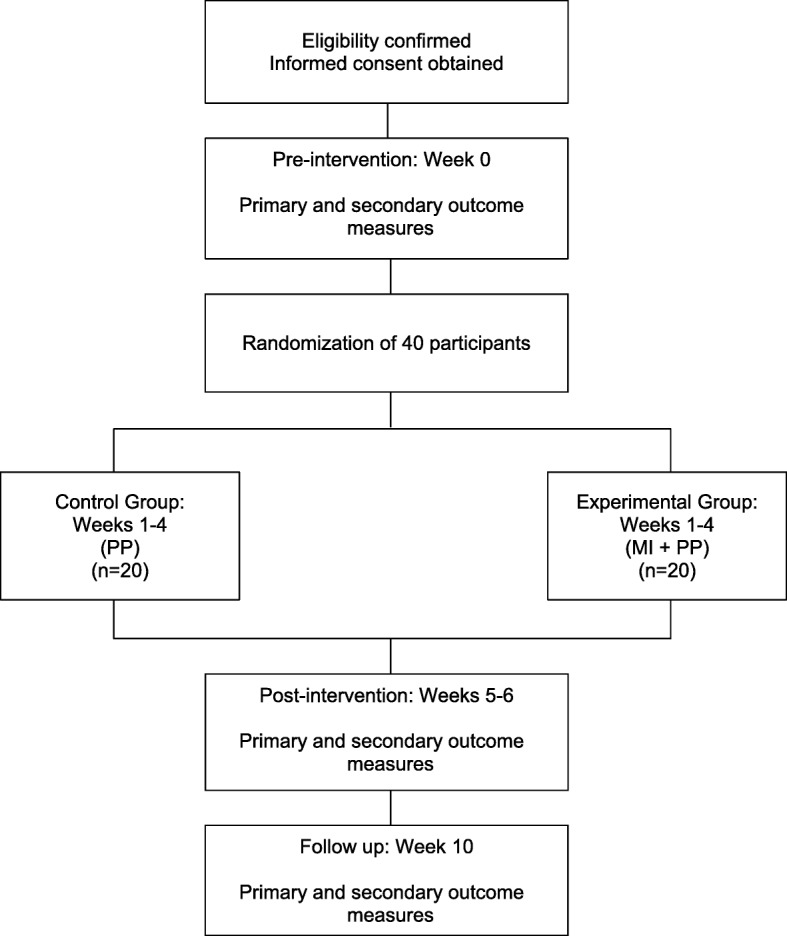
The schematic study design

This protocol will be applied on the day following initial assessment, executed in 12 training sessions of at most 90 min, three times a week for 4 weeks. Experimental group individuals will be instructed to continue to practice MI of gait in their daily lives during the training period. They will be instructed to fill in a diary with information on the quantity of days, number of repetitions and duration of the training performed at home. All the participants will be reassessed 1, 7 and 30 days after the last training session in terms of the kinematic variables of gait using the Qualisys Motion Capture System®, mobility using the TUG and EEG activity via Emotiv EPOC+. During the gait test, the patient will be instructed to walk as they did over the past days, since the beginning of the study participation. As the evaluator is blind to allocation groups, they will not know whether the patient is making corrections or not in the gait. The schematic study design is shown in Fig. 2.

### Data analysis

Data analysis will be conducted with the following variables: (1) Kinematic: speed (m/s), stride length (m), stance and swing time (s) of the most affected limb, hip range of motion, maximum hip extension during stance (°), maximum hip flexion during swing (°), maximum knee flexion during swing (°) and ankle dorsiflexion (°); (2) EEG activity: alpha (8–12 Hz)- and beta (13–30 Hz)-frequency bands; and (3) Mobility: average time to conduct the TUG.

Analysis of variance with repeated measures will be used to determine whether there are statistically significant differences between baseline, post training and monitoring measures of the CG and the EG.

Group descriptions will be presented as means and standard deviations. Intention-to-treat analysis will be conducted for dropout data, using the last available value to represent the absent assessment sessions.

## Discussion

The protocol aims to determine the effects of motor-imagery training on gait and the EEG activity of individuals with PD. This protocol was based on those of Santiago [19] and El-Whishy [18], but the type of observation, follow-up and training time were altered.

In Santiago’s protocol [19], the action was visualized using images of the sequential stages of gait. It is a static method that does not resemble the real pattern. The protocol, based on El-Whishy [18], involves observing the action using videos of normal gait and of a patient with PD, enabling better identification of gait changes and familiarizing individuals with their daily gait pattern, making the training more dynamic.

The second alteration is related to the lack of follow-up in El-Whishy’s protocol [18]. It is known that the presence of a follow-up is important in confirming the retention time of the observed effects. This protocol, which is different from the aforementioned, suggests a follow-up in order for these effects can be assessed.

The third and last alteration is related to the training used. Based on the study by Santiago [19] and El-Whishy’s protocol [18], it is suggested that patients with PD require more time to achieve favorable results. As such, this study proposed to use a longer training period in order to investigate whether the increase in MI training associated with PP is able to produce benefits superior to those of PP alone.

The methodological strength of the proposed protocol lies in the fact that it is a prospectively registered randomized controlled study. The study also includes random allocation and masking, in addition to an intent-to-treat analysis. The sample size was calculated to provide an adequate statistical base to identify intergroup differences in the primary outcome. This study has limitations, given that the participants and therapist cannot be blinded in complex interventions. Moreover, it is difficult to ensure that patients will imagine movement, that they will use this imagination in the kinesthetic modality and in the number of repetitions proposed.

In conclusion, the results of the present study may provide an important advance in neurological rehabilitation. An easy-access, low-cost intervention may help improve gait, EEG activity and mobility in individuals with PD.

## Additional file

Additional file 1: Standard Protocol Items: Recommendations for Interventional Trials (SPIRIT) 2013 Checklist: recommended items to address in a clinical trial protocol and related documents. (DOC 116 kb)

## Data Availability

Not applicable.

## Abbreviations

CG: Control group
CNPq: National Council for Scientific and Technological Development
EEG: Electroencephalographic
EG: Experimental group
LIAM: Laboratory of Intervention and Analysis of Movement
MI: Motor imagery
MIQ-R: Revised Movement Imagery Questionnaire
MMSE: Mini-Mental State Examination
PD: Parkinson’s disease
PP: Physical practice
TUG: Timed Up and Go Test
UFRN: Federal University of Rio Grande do Norte
